# The Clinical Link between Human Intestinal Microbiota and Systemic Cancer Therapy

**DOI:** 10.3390/ijms20174145

**Published:** 2019-08-25

**Authors:** Romy Aarnoutse, Janine Ziemons, John Penders, Sander S. Rensen, Judith de Vos-Geelen, Marjolein L. Smidt

**Affiliations:** 1 GROW-School for Oncology and Developmental Biology, Maastricht University Medical Center+, 6229 ER Maastricht, The Netherlands; 2 Department of Surgery, Maastricht University Medical Center+, 6202 AZ Maastricht, The Netherlands; 3 Department of Medical Microbiology, Maastricht University Medical Center+, 6202 AZ Maastricht, The Netherlands; 4 NUTRIM - School of Nutrition and Translational Research in Metabolism, Maastricht University Medical Center+, 6229 ER Maastricht, The Netherlands; 5 Department of Internal Medicine, Division of Medical Oncology, Maastricht University Medical Center+, 6202 AZ Maastricht, The Netherlands

**Keywords:** human intestinal microbiota, systemic cancer therapy, chemotherapy, immunotherapy, hormonal therapy, clinical relevance, baseline microbiota sampling, longitudinal microbiota sampling, 16S rRNA gene sequencing, metagenomic sequencing

## Abstract

Clinical interest in the human intestinal microbiota has increased considerably. However, an overview of clinical studies investigating the link between the human intestinal microbiota and systemic cancer therapy is lacking. This systematic review summarizes all clinical studies describing the association between baseline intestinal microbiota and systemic cancer therapy outcome as well as therapy-related changes in intestinal microbiota composition. A systematic literature search was performed and provided 23 articles. There were strong indications for a close association between the intestinal microbiota and outcome of immunotherapy. Furthermore, the development of chemotherapy-induced infectious complications seemed to be associated with the baseline microbiota profile. Both chemotherapy and immunotherapy induced drastic changes in gut microbiota composition with possible consequences for treatment efficacy. Evidence in the field of hormonal therapy was very limited. Large heterogeneity concerning study design, study population, and methods used for analysis limited comparability and generalization of results. For the future, longitudinal studies investigating the predictive ability of baseline intestinal microbiota concerning treatment outcome and complications as well as the potential use of microbiota-modulating strategies in cancer patients are required. More knowledge in this field is likely to be of clinical benefit since modulation of the microbiota might support cancer therapy in the future.

## 1. Introduction

The human microbiota is the collection of bacteria, archaea, viruses, and eukaryotic microorganisms that live in and on the human gastrointestinal tract, mucosae, and skin. The microbiome is the collective genome of the microbiota and encodes approximately 100-fold more genes than the human genome itself [1]. The majority of the microbiota resides in the gastrointestinal tract and belongs to the ‘intestinal microbiota’ or ‘gut microbiota’.

It has been established that cross-talk between the gut microbiota and the human host is essential for maintaining homeostasis and human health [2]. Therefore, it is not surprising that microbial dysbiosis has been shown to be associated with various metabolic and inflammatory diseases, such as ulcerative colitis, obesity, diabetes mellitus, and hypertension [3,4,5].

Next to the taxonomic composition of the gut microbiota, the intra- and inter-individual diversity of the microbial community are considered to be of great importance [3,4,6]. Microbial diversity can be quantified by means of two metrics: α-diversity and β-diversity. α-diversity describes the number (richness) and distribution (evenness) of taxa in a given sample [7]. Common indices to describe α-diversity are the Shannon index, Simpson index, and the Chao 1 index [7]. β-diversity defines the number of taxa shared between different samples and can be seen as a (dis)similarity score [7]. Generally, a healthy state is characterized by a species-rich, diverse, and stable microbiota, which fulfills various and complex metabolic roles [8].

In recent years, increasing evidence shows that the gut microbiota has an important role in carcinogenesis and the pathophysiology of human cancer. For instance, infection with *Helicobacter pylori* is considered to stimulate the development of gastric carcinoma by producing virulence factors and enhancing chronic inflammation and subsequent carcinogenesis [9]. Similarly, abundance of *Fusobacterium nucleatum* has been found to be increased in colorectal cancer and it is suggested that this bacterial species might be involved in intestinal tumorigenesis and modulation of the tumor microenvironment [10,11].

Interestingly, the involvement of the gut microbiota is not limited to gastrointestinal cancers. It has been suggested that gut bacteria affect the development of breast cancer through modulation of estrogen metabolism [12,13]. In line with this, it has been demonstrated that gut microbiota composition, as well as several functional features, differ between postmenopausal breast cancer patients and healthy controls [14]. Furthermore, Rajagopala et al. (2016) demonstrated that patients with leukemia already had reduced microbial diversity and dysbiosis at the time of diagnosis and could be distinguished from healthy controls based on their microbiota profiles [15].

While there are strong indications for the role of the gut microbiota in carcinogenesis, evidence concerning its role in the context of cancer treatment is scarce. Currently, most of the results concerning interactions between the gut microbiota and cancer therapy originate from in-vitro studies using culturing methods [16,17,18]. A comprehensive overview of clinical studies in this field of research is lacking.

**Figure 1:**
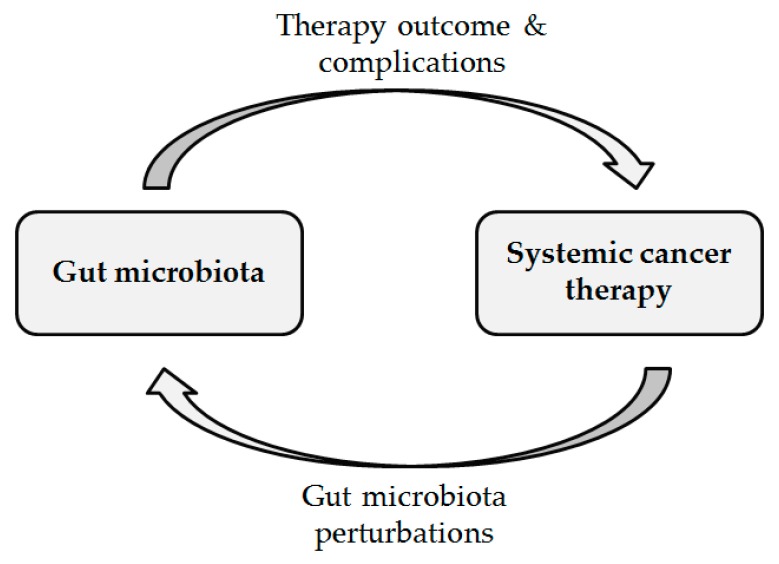
Overview of the main questions addressed in this review.

This systematic review summarizes clinical studies investigating the influence of the intestinal microbiota on systemic cancer therapy as well as the influence of systemic cancer therapy on the intestinal microbiota (Figure 1). We focused on chemotherapy, immunotherapy, and hormonal therapy. In addition, Table A2 in Appendix B provides an overview of important terms used in microbiota research. By providing a comprehensive overview of clinical studies on the interaction between the gut microbiota and systemic cancer therapy, this review will provide pivotal information on current gaps of knowledge and will facilitate the evidence-based design of future studies in this field.

## 2. Baseline Human Intestinal Microbiota Characteristics Are Associated with the Development of Complications and Systemic Cancer Therapy Outcome

### 2.1. Chemotherapy

Infectious complications are a common side effect of cancer therapy and have a considerable impact on patients’ prognosis and quality of life [19]. Research indicated that the development of chemotherapy-related infections might be associated with intestinal microbiota composition.

Galloway-Pena et al. (2016) demonstrated that baseline α-diversity was significantly lower in patients with acute myeloid leukemia (AML) suffering from infectious complications after induction chemotherapy compared to patients without infections [20]. Consequently, a lower microbial diversity before the start of chemotherapy might increase the risk for the development of infections, potentially as a result of a reduced colonization resistance. Additionally, the same group also analyzed stool temporal variability as indicator of microbial instability and its association with induction chemotherapy outcome [21]. Baseline samples were collected up to eight days before and 24 h following chemotherapy initiation. It was concluded that AML patients who developed an infection within 90 days post-neutrophil recovery had significantly higher microbial instability [21]. Moreover, patients developing an infection during induction chemotherapy had a significantly higher relative abundance of *Stenotrophomonas* [21]. Intra-patient α-diversity variability was not associated with response to chemotherapy. Multivariate regression analysis indicated that age, antibiotic type and duration or chemotherapy regime were not significantly correlated with intra-patient temporal variability [21]. In conclusion, baseline stool microbiota with low α-diversity, high temporal variability and increased potentially pathogenic *Stenotrophomonas* are linked to infectious complications during and after induction chemotherapy. Consequently, patients with a less diverse and less stable gut microbiota might be at higher risk to develop infections.

In a study among 28 patients suffering from non-Hodgkin lymphoma, eleven were reported to develop bloodstream infections (BSI) [22]. Principal coordinate analysis (PCoA) of fecal samples collected before start of the treatment demonstrated differences between patients with or without subsequent BSI [22]. This means that the overall microbial community structure (β-diversity) was already different at baseline and that this might be predictive for future development of BSI. Similar to the results of Galloway-Pena et al. (2016), it was also shown that α-diversity was significantly lower in fecal samples from patients who developed subsequent BSI [22]. Furthermore, abundance of several bacteria was altered in these patients (Table 1). In addition, it was tested whether relative abundance of specific microbes could be used to discriminate between patients who did or did not develop subsequent BSI. In this context, Barnesiellaceae (AUC = 0.94), *Christensenellaceae* (AUC = 0.86) and *Faecalibacterium* (AUC = 0.84), which were all reduced in patients with subsequent BSI, were found to be promising candidates [22]. Based on these results, it was concluded that patients having a high risk to develop BSI could potentially be identified based on their microbial profile prior to therapy initiation.

In contrast with this study, the development of diarrhea in patients with metastatic renal cell carcinoma (RCC) was not related to differences in α-diversity of the gut microbiota [23]. However, clustering of these patients based on relative abundance at genus level revealed a low-risk and a high-risk group. The high-risk group had a high abundance of *Bacteroides* (42%) and a low level of *Prevotella* (3%) [23]. In the low-risk group, the opposite pattern was apparent with 47% *Prevotella* and 13% *Bacteroides* [23]. This suggests that there might be an interaction between intestinal microbiota composition and VEGF-TKI-induced diarrhea.

### 2.2. Immunotherapy

Six articles were available describing the association between baseline human intestinal microbiota and immunotherapy outcome [24,25,26,27,28,29]. Patients received anti-PD-1 or anti-CTLA-4 therapy for either metastatic or unresectable melanoma, renal cell carcinoma (RCC), or non-small cell lung cancer (NSCLC). Four out of these studies analyzed fecal microbiota composition with metagenomic shotgun sequencing [24,25,26,28].

Matson et al. (2018) compared the baseline microbiota composition of 42 patients with metastatic melanoma that received anti-PD-1 (*n* = 38) or anti-CTLA-4 (*n* = 4) immunotherapy [24]. Baseline stool samples were collected prior to immunotherapy initiation. Sixteen patients showed a response following immunotherapy, whereas 26 patients did not respond. Intestinal microbiota analysis indicated that one operational taxonomic unit (OTU) belonging to the family of *Bifidobacteriaceae* was significantly more abundant in the responder group compared to the non-responder group. Another *Bifidobacteriaceae* OTU (559527) was borderline significantly (*p* = 0.058) more abundant [24]. Principal component analysis (PCA) showed a separation of responders and non-responders [24]. Furthermore, eight species were more abundant in the responder group: *Enterococcus faecium*, *Collinsella aerofaciens*, *Bifidobacterium adolescentis*, *Klebsiella pneumoniae*, *Veillonella parvula*, *Parabacteroides merdae*, *Lactobacillus* sp., and *Bifidobacterium longum*—whereas two were more abundant in the non-responder group: *Ruminococcus obeum* and *Roseburia intestinalis* (Table 1). As conclusions did not change when removing the four anti-CTLA-4 treated patients, these patients were retained in the analysis [24]. This means that the baseline composition of the intestinal microbiota in patients with metastatic melanoma was associated with therapeutic efficacy of anti-PD-1 therapy. *Bifidobacteriam longum* and multiple other bacteria may contribute to improved anti-tumor immunity in patients. In addition, the ratio between potential ‘beneficial’ and ‘non-beneficial’ OTU’s might be a strong predictor of clinical response to anti-PD-1 therapy. The authors concluded that a higher ratio between beneficial and non-beneficial OTUs may predict the most favorable clinical outcome [24].

Gopalakrishnan et al. (2018) compared the microbiota composition of 43 metastatic melanoma patients treated with anti-PD-1 therapy [25]. Baseline stool samples were collected prior to therapy initiation. Median time from initial fecal sampling and therapy initiation was nine days with a broad range between -481 and +14 days. There were 30 responders and 13 non-responders. Pre-treatment α-diversity was significantly higher (*p* < 0.01) in responders compared to non-responders [25]. In addition, patients with a higher α-diversity prior to anti-PD-1 therapy had a significantly prolonged progression-free survival (PFS) compared to patients with an intermediate (*p* = 0.02) or low (*p* = 0.04) α-diversity [25]. The β-diversity at family level between responders and non-responders visualized with PCoA showed a clustering of samples (*p* < 0.05). Linear discriminant analysis (LDA) demonstrated that *Clostridiales* and *Ruminococcaceae* were enriched in responders and *Bacteroidales* enriched in non-responders (*p* < 0.01). Pairwise comparison identified that the *Faecalibacterium* genus was significantly enriched in responders. Using whole metagenome sequencing (WMGS), *Faecalibacterium* sp., *Clostridium* sp., *Clostridiales*, *Eubacterium* sp., *Oscillibacter* sp., and *Ruminococcaceae* were found to be enriched in responders (*n* = 14). *Bacteroides thetaiotaomicron, Escherichia coli*, *Oxalobacter formigenes*, *Anaerotruncus colihominis*, and *Klebsiella variicola* were significantly enriched in non-responders (*n* = 11) [25]. Nineteen out of 39 patients had a high abundance of *Faecalibacterium*, accompanied by a significantly prolonged PFS compared to patients with lower abundance (*p* = 0.03). Twenty of 39 patients had a high abundance of *Bacteroidales*, accompanied by a shortened PFS compared to patients with a lower abundance (*p* = 0.05). Cox proportional hazard analysis demonstrated that α-diversity and the abundance of *Faecalibacterium* and *Bacteriodales* were significant strong predictors of response to anti-PD-1 therapy in metastatic melanoma patients [25]. Patients with a high α-diversity and abundance of *Ruminococcaceae* and *Faecalibacterium* were found to have an enhanced systemic and antitumor immune response mediated by increased antigen presentation and improved effector T-cell function. Conversely, patients with low α-diversity and high relative abundance of *Bacteroidales* had an impaired immune response [25].

Routy et al. (2018) analyzed 100 patients who received anti-PD-1 therapy for NSCLC (*n* = 60) or RCC (*n* = 40) [26]. Baseline fecal samples were collected before anti-PD-1 infusion. Since there were no statistically significant differences in gene count and metagenomic species before and during anti-PD-1 therapy samples, T1 samples (collected after two anti-PD-1 infusions) were used if baseline samples were not available. A significantly higher α-diversity (richness at gene count (*p* = 0.002) and metagenomic species level (*p* = 0.003)) of fecal samples was correlated with clinical response at six months, but not at three months after therapy initiation [26]. Response was defined as the absence of progression defined by the Response Evaluation Criteria in Solid Tumors (RECIST) [26]. By means of the RECIST criteria, tumor response can be graded as complete response (CR), partial response (PR), progressive disease (PD), or stable disease (SD) [30]. In addition, Routy et al. (2018) identified that for instance Firmicutes, *Akkermansia* and *Alistipes* were significantly associated with response (PR and SD) [26]. *Akkermansia muciniphila* was most significantly (*p* = 0.004) overrepresented at diagnosis in the feces of responders and patients with a PFS > 3 months after anti-PD-1 therapy initiation (*p* = 0.028). These results were independent of antibiotic use [26]. Moreover, several additional bacterial species were significantly increased or decreased in patients with a PFS > 3 months excluding those who took antibiotics (*n* = 78), see Table 1. Similar results were seen when all patients were included (*n* = 100). Comparable results were obtained in the cohort of NSCLC patients (*n* = 58). In particular, it was notable that when high levels of *Akkermansia muciniphila* were present in the feces, patients would later benefit from anti-PD-1 therapy [26].

Chaput et al. (2017) analyzed the predictive value of baseline fecal microbiota samples of 26 patients with metastatic melanoma receiving ipilimumab [27]. Baseline fecal samples were collected before the first ipilimumab infusion. PCA analysis at genera level (*p* = 0.0090), species level (*p* = 0.0050) or OTU level (*p* = 0.0080) indicated that metastatic melanoma patients could be clustered into groups with long-term versus poor clinical benefit, based on gut microbiota composition at baseline [27]. Main genera which contributed to this stratification were *Faecalibacterium, Gemmiger, Bacteroides*, and *Clostridium XIVa* [27]. Before treatment, patients with poor clinical benefit had a high proportion of *Bacteroides* (*p* = 0.034). The relative abundance of *Faecalibacterium, Clostridium XIVa*, and *Gemminger* was higher in patients with long term benefit [27]. Additionally, patients with higher levels of *Ruminococcus* and *Lachnospiraceae* (relatives of *Facealibacterium prausnitzii L2–L6*, *Gemmiger formicilis*, and *butyrate-producing bacterium SS2-1*) at baseline had an overall survival (OS) longer than 18 months. These results were independent of previous antibiotic use and antibiotic use did not influence baseline dominant microbiota [27].

Three clusters could be identified based on baseline microbiota composition at the genus level. The first cluster (*n* = 12) was enriched in *Faecalibacterium*, and other Firmicutes (unclassified *Ruminococcaceae, Clostridium XIVa*, and *Blautia*), had a longer PFS (*p* = 0.0039), OS (*p* = 0.051) and greater clinical benefit (*p* = 0.0017) compared to patients in the second cluster with baseline samples enriched in *Bacteroides* (*n* = 10). The third cluster of patients was enriched in *Prevotella* (*n* = 4), but was not included in the analysis due to the low number [27]. It was further shown that patients with baseline samples enriched in Firmicutes were more prone to develop colitis (*p* = 0.009), while patients with enhanced baseline Bacteroidetes did not develop colitis (*p* = 0.011) [27]. These findings indicate that gut colonization with Firmicutes is associated with a better anti-cancer response and colitis in metastatic melanoma patients that will be treated with ipilimumab. On the other hand, gut colonization with Bacteroidetes appears to be associated with a poor response without colitis [27].

Frankel et al. (2017) collected baseline fecal samples of 39 unresectable or metastatic melanoma patients before treatment with anti-PD-1 or anti-CTLA-4 (ipilimumab (I), nivolumab (N), ipilimumab + nivolumab (IN), or pembrolizumab (P)) [28]. Response was quantified by means of the RECIST criteria and was defined as stable or responsive disease. Metagenomic shotgun sequencing indicated that responders (*n* = 24) were significantly enriched with *Bacteroides caccae* (*p* = 0.032) and *Streptococcus parasanguinis* (*p* = 0.048) [28]. In the IN + N group there were 16 responders and eight non-responders [28]. Within this group, responders treated with IN (*n* = 16) and N (*n* = 1) were significantly enriched with *Faecalibacterium prausnitzii* (*p* = 0.032), *Holdemania filiformis* (*p* = 0.043), and *Bacteroides thetaiotamicron* (*p* = 0.046). Responders treated with P had significantly higher levels of *Dorea formicigenerans* (*p* = 0.045). The P group contained six responders and seven non-responders [28].

Interestingly, overall microbial diversity was not significantly different between responders and patients with progressive disease [28]. Overall, this study identified specific gut microbiota species associated with response to anti-PD-1 and anti-CTLA-4 therapy.

Dubin et al. (2016) correlated fecal microbiota composition with subsequent colitis development in 34 patients with metastatic melanoma to be treated with ipilimumab [29]. In general, fecal samples were obtained from patients before the first dose of ipilimumab (30/34). Ten patients with metastatic melanoma developed colitis between 13 and 57 days after ipilimumab initiation. Colitis-free patients (*n* = 24) had an increased relative abundance of Bacteroidaceae, Bacteroides, Barnesiellaceae, unclassified Barnesiellaceae, Rikenellaceae, unclassified Rikenellaceae, Bacteroidetes, Bacteroidia, and Bacteroidales. Patients that developed colitis (*n* = 10) had a decreased relative abundance of Bacteroidetes in fecal samples collected before ipilimumab infusion [29]. Based on this, the authors concluded that increased fecal abundance of Bacteroidetes, Bacteroidaceae, Rikenellaceae, and Barnesiellaceae correlated with a reduced risk to develop ipilimumab-induced colitis [29].

### 2.3. Hormonal Therapy

To the best of our knowledge, clinical studies investigating the association between baseline human intestinal microbiota and the outcome of hormonal therapy have not been reported so far.

## 3. Human Intestinal Microbiota Changes during Systemic Cancer Therapy

### 3.1. Chemotherapy

Several studies investigated the effect of systemic cancer therapy on gut microbiota composition in different types of cancer. These studies included gastrointestinal and non-gastrointestinal cancers as well as different chemotherapeutic agents and treatment settings.

In patients with neuroendocrine tumors (NET), it was observed that systemic chemotherapy increased the concentration of *Faecalibacterium prausnitzii* in patients with midgut NET [31]. While this study used fluorescent in situ hybridization (FISH) targeting selected species only, more recent articles use sequencing-based approaches in order to extensively profile the bacterial species composition.

Using sequencing of the 16S rRNA gene, Montassier et al. (2014) observed a remarkable shift of the intestinal microbiota composition during five day high-dose chemotherapy as conditioning regimen for bone marrow transplantation [6]. More precisely, there was a significant reduction in the observed microbial richness (number of bacterial taxa), estimated microbial richness (Chao1 index), as well as microbial diversity (Shannon index), indicating a significant reduction in α-diversity due to chemotherapy (*p* < 0.001) [6]. Furthermore, PCoA showed a clear separation of pre-chemotherapy and post-chemotherapy samples (*p* < 0.001) [6]. Thus, it can be concluded that high-dose chemotherapy induced a marked decrease in overall microbial diversity and shifted the microbial community structure. On the phylum level, abundance of Bacteroidetes and Proteobacteria was increased, while Firmicutes and Actinobacteria were decreased [6]. On the genus level, *Bacteroides* (*p* = 0.0008) and *Escherichia* (*p* = 0.008) were significantly higher in the post-chemotherapy samples compared to pre-chemotherapy samples. On the other hand, *Blautia* (*p* = 0.008), *Faecalibacterium* (*p* = 0.04), *Roseburia* (*p* = 0.008), and *Bifidobacterium* (*p = 0.04*), which are considered health promoting and anti-inflammatory bacteria, were decreased after chemotherapy [6]. Furthermore, there was a statistically significant shift from Gram-positive bacteria to Gram-negative bacteria during chemotherapy (*p* < 0.001) [6]. Interestingly, this study also described that several less abundant bacterial genera appeared after chemotherapy treatment [6]. A similar observation was described by Zwielehner et al. (2011) [32].

In a subsequent study, Montassier et al. (2015) verified the previously described results concerning microbial diversity and differences at the phylum level [33]. Additionally, abundance of *Ruminococcus*, *Oscillospira*, *Blautia*, *Lachnospira*, *Roseburia*, *Dorea*, *Coprococcus*, *Anaerostipes*, *Clostridium*, *Collinsella*, *Adlercreutzia*, and *Bifidobacterium* were decreased after chemotherapy (*p* < 0.05) while the abundance of *Citrobacter*, *Klebsiella*, *Enterococcus*, *Megasphaera*, and *Parabacteroides* was increased (*p* < 0.05) [33]. Besides these profound changes in intestinal microbiota composition, shifts in microbial functions were observed by means of a computational approach. Amino acid metabolism (*p* = 0.0004), nucleotide metabolism (*p* = 0.0001), energy metabolism (*p* = 0.001), as well as metabolism of cofactors and vitamins (*p* = 0.006) were depleted in samples collected after chemotherapy compared to samples collected before chemotherapy [33]. Concurrently, signal transduction (*p* = 0.0002), xenobiotics biodegradation (*p* = 0.002), and glycan metabolism (*p* = 0.0002) were enhanced [33]. Furthermore, several other metabolic pathways, amongst others pathways involved in bacterial motility, virulence, and epithelial repair were altered after chemotherapy [33].

Galloway-Peña et al. (2016) observed similar dramatic changes in the intestinal microbiota composition in AML patients during induction chemotherapy [20]. Using 16S rRNA gene sequencing, they identified a statistically significant progressive decrease in overall microbial diversity as well as decreased abundance of the anaerobic genus *Blautia* [20]. On the other hand, chemotherapy caused increased abundance of *Lactobacillus* [20]. Interestingly, chemotherapy also increased the occurrence of a phenomenon called intestinal domination, which means that more than 30% of the intestinal bacteria belong to a single taxon. After completion of chemotherapy, 50% of the domination events was caused by opportunistic pathogenic bacteria, known to induce bacteremia (e.g., *Staphylococcus*, *Enterobacter*, *Escherichia*). Before chemotherapy, this was only 20% [20]. In addition, induction chemotherapy resulted in a high variation in temporal stability, as assessed by calculating the coefficient of variation (CV) of the Shannon index [21]. Furthermore, high intra-patient temporal instability was associated with increased abundance of opportunistic pathogenic genera [21]. High CV values were positively correlated with pathogenic genera such as *Staphylococcus* and *Streptococcus* and negatively associated with the non-pathogenic *Akkermansia* [21]. Thus, a high relative abundance of *Akkermansia*, *Subdilogranulum*, and *Pseudobutvrivibrio* was associated with a more stable microbiome during induction chemotherapy. Potentially pathogenic bacteria such as *Streptococcus* and *Staphylococcus* were more abundant in patients with a more variable microbiome [21].

Different studies focused on the effect of chemotherapy on gut microbiota composition in gastrointestinal cancers. For instance, Sze et al. (2017) collected pre- and post-treatment fecal samples of 26 colorectal cancer (CRC) patients treated with different types of chemotherapy [34]. A significant change in community structure (β-diversity) between pre- and post-treatment samples was observed (*p* = 0.005). Using random forest models, collections of OTUs were identified that differentiated between pre- and post-treatment samples (AUC 0.82–0.98) [34]. However, no significant change in α-diversity between pre- and post-treatment samples was identified [34]. The authors concluded that the community structure was affected by the treatment, but the effect of treatment was not consistent across patients [34]. No subgroup analysis was performed for these very heterogeneous small groups receiving chemotherapy or chemoradiation. Next, Sze et al. constructed a random forest model using CRC patients (*n* = 94) and healthy controls (*n* = 172) in order to define a normal gut microbiota profile. Afterwards, it was indicated that gut microbiota composition of 19 out of 26 treated CRC patients (73%) shifted towards this normal profile (*p* = 0.001) [34]. Hence, it was concluded that the treatment induced a shift towards a microbial profile that has great similarity to the gut microbiota of healthy participants [34]. These results are contradictory to the studies described before, which indicated deterioration of the gut microbiota instead of improvement.

Youssef et al. (2018) collected fecal samples of 20 treated patients with gastrointestinal neoplasms and 13 healthy controls [35]. Gastrointestinal neoplasms included neoplasms of the stomach (*n* = 6), small intestine (*n* = 1), or rectum (*n* = 13). Treatment included chemotherapy and/or radiotherapy [35]. 16S rRNA gene sequencing indicated that at the genus level, the α-diversity, genus richness, and β-diversity did not significantly differ between controls (*n* = 13) and non-treated patients (*n* = 43) compared to treated patients (*n* = 20). Patients treated with chemotherapy and/or radiotherapy had a significantly higher relative abundance of Lactobacillaceae and *Lactobacillus* compared to untreated patients with gastrointestinal neoplasms. In comparison to healthy controls, treated patients had a significantly lower relative abundance of Bifidobacteriaceae *Ruminiclostridium*, *Lachnoclosteridium*, and *Oscillibacter* [35].

Similarly, Deng et al. (2018) compared fecal microbiota composition of 14 CRC patients treated with chemotherapy with 33 healthy controls [36]. Chemotherapy consisted of the 5-fluoruouracil (5-FU) precursor tegafur and oxaliplatin. Compared to healthy controls, *Veillonella* at the genus level and *Veillonella dispar* at the species level were only present in CRC patients. *Prevotella copri* and *Bacteroides plebeius* were enriched in patients treated with chemotherapy compared to controls [36].

In a cohort of patients with different cancer types, Zwielehner et al. (2011) indicated that species richness within the *Clostridium* cluster *IV* was remarkably reduced immediately after chemotherapy, but recovered within 5–9 days after chemotherapy [32]. Likewise, total bacterial abundance declined after chemotherapeutic treatment (*p* = 0.037) and was also restored within a few days [32]. Next to *Clostridium* cluster *IV*, the bacteria found to be affected most by chemotherapy were *Bacteroides*, *Bifidobacteria*, as well as *Clostridium* cluster *XIVa* [32].

In a similar study, it was demonstrated that cancer patients receiving chemotherapy for different cancer types were characterized by a decreased relative abundance of *Lactobacillus spp*., *Bacteroides spp*., *Bifidobacterium spp*., and *Enterococcus spp*. when compared to healthy controls [37]. Increased relative abundance was found for *Escherichia coli* and *Staphylococcus spp*. [37]. These findings were complemented with the observation that the abundance of *Escherichia coli* gradually increased during chemotherapy, while the initial increase of *Lactobacillus spp*. was followed by a decreased abundance after 10 days [37].

Besides, some studies investigated the effect of chemotherapy on the gut microbiota in pediatric patients. In this context, Wada et al. (2010) reported that the start of chemotherapy induced an increase of the facultative anaerobic Enterobacteriaceae in children with malignancies [38].

In addition, another study with pediatric AML patients revealed that there was a considerable decrease in bacterial diversity during chemotherapy treatment, which restored quickly after chemotherapy [39]. Furthermore, the total number of bacteria was found to be significantly reduced in patients during treatment but resembled the bacterial count in healthy samples six weeks after the last chemotherapy cycle [39]. This reduced number of bacteria was caused by a 3000–6000-fold decrease of the anaerobic *Bacteroides*, *Clostridium* cluster *XIVa*, *Faecalibacterium prausnitzii*, and *Bifidobacterium*. Interestingly, only *Clostridium XIVa* and *Faecalibacterium prausnitzii* levels were restored six weeks after treatment [39]. The number of aerobic enterococci was significantly higher in patients compared to healthy controls, while the number of streptococci was 100–1000 fold decreased in patient samples [39]. Of note, the disturbed balance marked by a dramatic reduction of anaerobic bacteria and increased enterococci levels might have negative consequences for the risk of infection and colonization with potentially pathogenic bacteria [39].

On the contrary, Rajagopala et al. (2016) indicated that there was no difference in microbial diversity before and during induction chemotherapy in patients with pediatric and adolescent ALL [15]. It was also shown that microbial diversity was significantly higher during maintenance chemotherapy compared to baseline, which is not in line with the results described above [15,39]. Of note, this study also demonstrated that microbial dysbiosis was already present at the time of diagnosis. By comparing ALL patients and their healthy siblings, it was found that all patients were characterized by decreased diversity and decreased relative abundance of Lachnospiraceae (including Clostridium XIVa, IV) *Roseburia, Anaerostipes, Coprococcus*, and *Ruminococcus 2*. [15]. Bacteroides occurrence was increased in these patients [15]. In view of the fact that ALL patients suffer from an impaired immune system at the time of diagnosis [40], it might be suggested that the increasing microbial diversity during therapy might be interpreted as an indication for the anti-cancer effect of the therapy.

### 3.2. Immunotherapy

Six articles of five human clinical studies were identified that described human intestinal microbiota changes during immunotherapy assed by longitudinal sampling [25,26,27,28,31,41]. Patients received anti-PD-1, anti-CTLA-4 or interferon alpha-2b therapy for either metastatic or unresectable melanoma, renal cell carcinoma (RCC), non-small cell lung cancer (NSCLC), or neuroendocrine tumors (NET).

Routy et al. (2018) collected longitudinal fecal samples of 32 patients that received two months anti-PD-1 therapy for NSCLC (*n* = 15) or RCC (*n* = 17) [26]. Feces were collected before start of the treatment, as well as after the 2nd (one month), 4th (two months), and 12th (six months) anti-PD-1 infusion. The stool α-diversity (richness at metagenomic species (MGS) level) increased. Stool richness at MGS level increased more in RCC patients (*p* = 0.033) compared to NSCLC and RCC patients together (*p* = 0.046). None of the 32 patients received antibiotics. After two months, anti-PD-1 therapy, the following bacteria were enriched: *Candidatus Alistipes marseilloanorexicus*, *Clostridium scindens*, *Eubacterium sp*., *Clostridium sp*., *Streptococcus salivarius*, *Clostridiales*, and *Eubacterium eligens* [26].

Chaput et al. (2017) collected longitudinal fecal samples of 26 patients with metastatic melanoma [27]. Patients received four cycles of ipilimumab every three weeks. Fecal samples were collected before the first ipilimumab infusion (*n* = 26), before each following infusion (V2: *n* = 14, V3: *n* = 15, V4: *n* = 13) and 3 weeks after the last infusion (*n* = 4). It was observed that the phyla Firmicutes and Bacteroidetes remained stable during treatment with ipilimumab. Additionally, Shannon and Simpson α-diversity indices did not change during ipilimumab treatment, thereby suggesting that ipilimumab treatment did not modify the gut microbiota [27]. However, it should be noted that the number of fecal samples analyzed decreased to four over time [27]. While there was no direct effect of ipilimumab on the gut microbiota in this study, the authors reported changes in gut microbiota composition at the time of colitis occurrence during ipilimumab treatment. Therefore, fecal samples of seven patients with colitis were collected and compared with baseline samples. At family level (*p* = 0.0049) as well as at genus level (*p* = 0.0059), significant differences in microbiota composition were observed. Relative abundance of seven dominant genera (*Ruminococcus*, *Lachnospiracea incertae sedis*, *Blautia*, *Clostridium IV*, *Eubacterium*, unclassified *Lachnospiraceae*, and *Pseudoflavonifracto*) was significantly reduced in metastatic melanoma patients with ipilimumab-induced colitis [27]. They all belong to the Firmicutes phylum. Furthermore, 18 other bacteria, mostly Firmicutes, were significantly reduced (Table 2). Ipilimumab-induced colitis was also associated with lower α-diversity [27]. However, these microbial perturbations were most likely caused by the colitis instead of the therapy itself.

Prior to this study, Vetizou et al. (2015) already published results concerning gut microbiota composition in patients with metastatic melanoma before (*n* = 19) and after (*n* = 18) treatment with ipilimumab [41]. These patients were later also described in the article of Chaput et al. (2017) in relation to ipilimumab-induced colitis [27]. Patients were divided into three clusters based on genus composition. Cluster A was enriched in *Alloprevotella* and *Prevotella;* cluster B was enriched with relatives of *Prevotella copri, Bacteroides sp. CCUG 39913, Barnesiella intestinohominis YIT 11860*, and *Parabacteroides distasonis M86695* and cluster C was enriched in *Bacteroides salyersiae WAL 10018*, *Bacteroides acidifaciens AB021157*, and *Bacteroides uniformis JCM 5828T* [41]. During ipilimumab treatment, the proportion of patients in cluster C increased (*p* = 0.05) whereas it decreased in cluster B (*p* = 0.007) [41]. Interestingly, it has been shown that tumors in mice treated with ipilimumab respond better to fecal microbiota transplantation (FMT) of cluster C patients compared to FMT with cluster B enterotypes. This suggests that ipilimumab might modify the enterotype to the more favorable cluster C [41].

Additional to the studies of Routy, Chaput, and Vetizou, three studies performed longitudinal fecal sampling of a limited number of melanoma patients treated with immunotherapy. Gopalakrishnan et al. (2018) tested the stability of the gut microbiome during anti-PD-1 therapy in only three patients. Median time to repeat collection was 49 days (31–78 days) after initial sampling. They concluded that the α-diversity and microbiome composition at the order level was relatively stable during longitudinal sampling [25]. On the contrary, Frankel et al. (2017) performed longitudinal sampling, within one month after therapy initiation, of five patients (four responders and one patient with progressive disease) who received ipilimumab with nivolumab (*n* = 4) or pembrolizumab (*n* = 1) [28]. They concluded that specific gut microbiota abundances changed, but that these numbers were too small to draw conclusions [28]. In 2012, Dörffel et al. collected fecal samples in 11 patients with NET before and during interferon alpha-2b therapy. After four weeks of therapy, they observed by using FISH that interferon alpha-2b therapy was able to increase the concentration of *Faecalibacterium prausnitzii* to almost normal levels [31].

### 3.3. Hormonal Therapy

Currently, only two studies are available that investigated human intestinal microbiota changes during hormonal therapy [31,42].

Dörffel et al. (2012) collected fecal samples of 27 patients receiving somatostatin analogs for NET. It was observed that somatostatin analogs had no influence on the abundance of specific bacterial groups in these patients [31].

Sfanos et al. (2018) compared intestinal microbiota of patients with prostate cancer treated with androgen axis-targeted therapies compared to no hormonal medication use [42]. Androgen axis-targeted therapies included treatment with gonadotropin-releasing hormone (GNRH) (*n* = 5) or androgen receptor axis-targeted therapies (ATT) (*n* = 9). The group without hormonal medication included healthy controls (prostatic hyperplasia, *n* = 6), benign tumors (negative biopsy for prostate cancer, *n* = 3), and prostate cancer patients without therapy (*n* = 7). This study indicated no significant difference in α-diversity between prostate cancer patients treated with or without hormonal medication. The β-diversity was smallest within the ATT group compared to GNRH and the group without hormonal medication. The greatest β-diversity was seen between the ATT and the no medication group [42]. Together, these results indicate that the gut microbiota was most similar within the group of patients receiving ATT, while their microbiota was most dissimilar to that of the no medication group. Furthermore, ATT seemed to induce a low β-diversity. In the fecal samples of men taking oral ATT (*n* = 9) compared to no medication use (*n* = 16), several bacteria were significantly altered at species and/or family level (Table 3) [42]. In addition, it was confirmed that *Akkermansia muciniphila* was significantly more prevalent in men taking oral ATT, using quantitative polymerase chain reaction (qPCR) [42]. As indicated in Table 3, abundance of several bacteria at the species and family level was altered in men taking oral GNRH (*n* = 5) when compared to the group without use of hormonal medication (*n* = 16) [42].

## 4. Discussion

Awareness of the interaction between the human intestinal microbiota and systemic cancer therapy is increasing and results gained in this field of research potentially have considerable clinical implications. This review provided a detailed overview about all clinical studies describing the association between baseline intestinal microbiota and systemic cancer therapy as well as the influence of systemic cancer therapy on gut microbiota composition. We focused on systemic cancer therapy with chemotherapy, immunotherapy, and hormonal therapy.

### 4.1. Baseline Human Intestinal Microbiota Is Associated with the Development of Complications and Systemic Cancer Therapy Outcome

It became evident that baseline microbiota composition is associated with the development of (infectious) complications as well as with the outcome of systemic cancer therapy.

In the context of chemotherapy, research mainly focused on the association between baseline human intestinal microbiota composition and the development of chemotherapy associated complications, such as infections or diarrhea. It seems that patients with a particular intestinal microbiota are more prone to develop infections, likely as a result of a reduced colonization resistance, and that a beneficial intestinal microbiota might be protective. This is particularly interesting, since early identification of patients at risk for the development of complications would enable targeted interventions and the prevention of infectious complications in the future. Generally, gut microbiota composition of patients with infectious complications was characterized by reduced microbial diversity and increased microbial instability. Furthermore, *Stenotrophomonas* and *Bacteroides* were found to be increased, while *Barnesiellaceae*, *Christensenellaceae*, *Faecalibacterium*, and *Prevotella* were reduced. This suggests that these characteristics might be useful to identify patients at risk to develop subsequent infections.

To our knowledge, there is currently no clinical study published investigating the effect of the intestinal microbiota on chemotherapy efficacy. However, results from in-vitro studies strongly suggest an interaction between the gut microbiota and chemotherapy. For instance, it has been shown that the addition of *Lactobacillus plantarum* supernatant potentiates the therapeutic effect of 5-fluorouracil (5-FU) in chemoresistant cells [16]. In line with this, administration of an antibiotic cocktail markedly diminished the antitumor efficacy of 5-FU in mice [43]. Considering the complexity of the interaction, various metabolic pathways might be involved in microbial metabolism of chemotherapeutic drugs. The field of pharmacomicrobiomics focuses on unravelling these interactions between drugs and the human microbiome [44].

In this context, Alexander et al. (2017) suggested the TIMER mechanistic framework (translocation, immunomodulation, metabolism, enzymatic degradation, reduced diversity) to describe the mechanisms through which the gut microbiota might modulate chemotherapy treatment [45]. According to this concept, bacterial translocation might be facilitated by chemotherapeutic drugs which damage the intestinal barrier [45]. Subsequently, intestinal bacteria or their products can shape the chemotherapy-induced immune response by immunomodulation [45]. In support of this, Viaud et al. (2013) described that the intestinal microbiota influences the anticancer immune effects of cyclophosphamide by modulation of T-helper cells [17].

The most direct effect of the gut microbiota on drug metabolism is through metabolism and enzymatic degradation. Several gut bacteria-derived enzymes metabolize chemotherapeutic drugs and their metabolites, thereby modulating efficacy as well as toxicity [44,45]. Zimmermann et al. (2019) indicated that the orally administered capecitabine, cyclophosphamide, melphalan, and paclitaxel can be metabolized by specific bacterial strains [46]. A further example for direct microbial metabolism of chemotherapeutic drugs is a thymidine phosphorylase encoded by *Mycoplasma hyorhinis*. Activity of this enzyme has been shown to reduce the cytotoxic activity of several pyrimidine nucleoside analogues In contrast, the same thymidine phosphorylase enhanced cytotoxicity of capecitabine, probably by converting the pro-drug into the cytotoxic 5-FU [47].

Another factor with a considerable impact on cancer therapy outcome is chemotoxicity. High chemotoxicity often results in dose reduction or premature termination of the therapy, thereby severely limiting effectivity. In the case of the chemotherapeutic drug irinotecan, it is well described that microbial metabolism enhances chemotoxicity. It has been indicated that the bacterial enzyme β-glucuronidase reactivates previously detoxified SN-38G into the active metabolite SN-38, leading to severe toxicity in the gut [48]. In support of this, targeted inhibition of bacterial β-glucuronidase has been shown to alleviate gastrointestinal toxicity in mice [49].

Lastly, reduced microbial diversity and ecological variation might also affect the chemotherapy response of the host [45]. As shown in the present review, chemotherapy induces changes in gut microbiota composition and diversity. As a result, dysbiosis and overgrowth of potentially pathogenic bacteria might also have negative consequences for the treatment response. To conclude, it can be stated that we are currently only beginning to understand the whole biological complexity of microbiota-chemotherapy interactions.

A limited number (*n* = 6) of recently published articles describe the association of baseline human intestinal microbiota with immunotherapy outcome. The results of these studies suggest that a diverse and specific human intestinal microbiota (enriched in *B. longum* [24], *Faecalibacterium* [25,27,28], and *A. muciniphila* [26] and a reduced number of Bacteroidales [25] and Bacteroides [27]) stimulates and trains the immune system. This might result in increased antigen presentation, an improved effector T-cell function (increased CD4 +, CD8+ T-cells) and lower levels of regulatory T-cells [25]. An active and well developed immune system stimulates beneficial T-cell activation and consequently a diverse repertoire of T-cells [50]. Subsequently, this diverse pool of beneficial T-cells will be able to combat cancer cells by expressing multiple PD-1 receptors. PD-1 promotes apoptosis, reduces suppressive T-cells, and stimulates inflammation, resulting in an increased tumor response as well as inflammatory side effects like colitis. Consequently, it might be speculated that tumors had to be better developed and probably need multiple keys (PD-L1′s) in order to lock all the PD-1 receptors and to escape this efficient immune system. Based on this, it can be concluded that a more diverse T-cell repertoire, stimulated by a diverse intestinal microbiota, might inhibit tumor growth besides tumor suppressive effects of anti-PD-1 therapy. This theoretical basis evokes the question whether the immune modulatory effect of immunotherapy can be potentiated by the action of specific gut bacteria.

Results from mouse experiments provided further evidence for the immune modulatory effects of the intestinal microbiota. By transplanting baseline fecal microbiota of responder and non-responder patients to germ-free and tumor bearing mice, it was revealed that the clinical response was repeated in the majority of mice [24,25,26]. Moreover, the immune stimulatory effect of anti-CTLA-4 blockade reactivated T-cells, which resulted in anti-cancer response [27], but also immune-mediated colitis [27,29]. Bacteroidetes seemed to be associated with this clinical presentation [27,29]. Bacteroidetes could stimulate differentiation of regulatory T-cell [51] and consequently suppress the immune systems anti-cancer potency on the one hand and reduce colitis on the other hand.

The above presented mechanisms and interpretations indicate that the intestinal microbiota stimulates the immune system via multiple pathways. This might suggest that the addition of immunomodulation by microbiota modulation could be much more efficient compared to immunotherapy alone, since simultaneous activation of multiple tumor-suppressing pathways will inhibit tumor growth in a more efficient way.

Currently, there are no studies available that describe the role of the human intestinal microbiota in hormonal therapy, which is administered in hormone related malignancies like breast cancer, prostate cancer, and ovarian cancer. Since these are common malignancies with high morbidity, research is urgently required to investigate if baseline human intestinal microbiota is associated with hormonal therapy outcome.

### 4.2. Human Intestinal Microbiota Changes during Systemic Cancer Therapy

By definition, systemic cancer therapies affect the whole body. Therefore, it is not surprising that several studies investigated the effect of systemic cancer therapy on intestinal microbiota composition and that dramatic changes were reported.

Different studies investigated chemotherapy-induced changes of human gut microbiota composition by collecting fecal samples before and during chemotherapy. While only one study reported increased α-diversity during chemotherapy compared to baseline [15], the majority of the studies reported a chemotherapy-induced decrease of microbial diversity [6,20,33,39]. Furthermore, we identified key species which were shown to be affected in several studies. Abundance of Proteobacteria and Staphylococcus was found to be elevated due to chemotherapy. On the other hand, the phyla Firmicutes and Actinobacteria seemed to be negatively influenced by chemotherapy, leading to decreased levels of these bacteria. More specifically, *Blautia*, *Roseburia*, *Bifidobacterium*, as well as *Clostridium* cluster *IV* and *XIVa* were consistently found to be decreased during chemotherapy. For *Lactobacillus*, *Bacteroides*, *E.coli*, and *Faecalibacterium prausnitzii* the results were divergent, meaning that some studies reported increased abundance while others showed the opposite.

Interestingly, the bacteria found to be reduced during chemotherapy are prominent short chain fatty acid (SCFA) producing bacteria. SCFA are produced by microbial fermentation of non-digestible carbohydrates and are considered to fulfill a crucial role in colonic health. In particular, butyrate is essential for gut barrier integrity, since it serves as energy source for colonocytes [52]. Moreover, in-vitro studies showed that SCFA regulate the expression of tight-junction proteins and mucins [52]. In addition, SCFA have been shown to have potent anti-inflammatory as well as direct anti-carcinogenic effects [52]. Therefore, it might be suggested that the observed decrease in SCFA producing bacteria during chemotherapy might also have consequences for colonic SCFA concentrations and subsequently for the development of colonic inflammation and anti-cancer efficacy of the therapy.

Next to SCFA, there are also other metabolites of the gut microbiota that fulfill crucial physiological roles. In this context, secondary bile acids, branched chain fatty acids, as well as amino acids are repeatedly suggested as key metabolites [53]. The gut microbiota produces secondary bile acids by converting primary bile acids which were produced in the liver [54]. Mikó et al. (2018) showed that the secondary bile acid lithocholic acid inhibited cancer cell proliferation, tumor infiltration, as well as metastasis formation and improved the anti-tumor immune response [55]. Very recently, Colosimo et al. (2019) applied large-scale functional screening of molecules produced by gut bacteria in order to identify bacterial metabolites agonizing G-protein-coupled receptors (GPCRs) [56]. Amongst others, they identified phenylpropanoic acid, the amino acid cadaverine as well as the branched-chain fatty acid 12-methyltetradecanoic acid as promising molecules capable of modulating human signaling pathways through GPCR agonism [56]. Furthermore, microbial metabolites might also play a role in the indirect modulation of drug response [44].

Another interesting result of the current overview about changes in gut microbiota composition during chemotherapy is that specific bacteria might be more vulnerable to chemotherapy, compared to others. This might facilitate colonization with potentially pathogenic bacteria, such as Staphylococcus and many species belonging to the Proteobacteria phylum. As described by Zwielehner et al. (2011) [32] and Montassier et al. (2014) [6], elimination of specific bacteria might also lead to the appearance of less abundant bacterial genera. This phenomenon can be characterized by the term ‘functional response diversity’ which describes the different sensitivity of species to changes in the ecosystem [8]. This, in combination with the reduction of potentially health-promoting bacteria, might lead to severe dysbiosis in patients during chemotherapy, with possible negative consequences for chemotoxicity and treatment outcome.

In the field of immunotherapy, six studies collected longitudinal samples to determine human intestinal microbiota changes during immunotherapy with anti-PD-1, anti-CTLA-4 or interferon alpha-2b. Longitudinal microbiota sampling during four cycles of immunotherapy showed increases of microbiota richness and specific genera in one study [26], but did not affect microbiota diversity and the abundance of Firmicutes and Bacteroides in another [27]. In this study, only patients who developed colitis showed a reduced diversity and a significant difference in bacteria belonging to the Firmicutes phylum [27]. Vetizou et al. (2015) [41], who used the same patient population as described by Chaput et al. (2017) [27], observed microbiota changes during ipilimumab treatment.

Mechanisms by which immunotherapy influences intestinal microbiota composition are sparsely studied and are mainly based on mouse studies [57]. There are indications that anti-PD-1 therapy stimulates T-cell responses against intestinal bacteria and consequently improves cancer cell surveillance and detection [26,58]. In mice, anti-CTLA-4 therapy promoted pro-inflammatory pathways and induced intestinal epithelial cell death and proliferation. In patients, anti-CTLA-4 therapy led to microbial dysbiosis at the genus level by a not yet fully explored mechanism. Dysbiosis promoted T-helper 1 and dendritic cell maturation in humans. This consequently affected anti-cancer therapy efficacy [41].

Based on the available studies and proposed mechanisms, no strong conclusions could be drawn. Additional clinical research should reveal if immunotherapy influences the human intestinal microbiota composition and its relation with anti-cancer therapy efficacy. If immune modulatory effects could be attributed to the intestinal microbiota composition changes, future systemic cancer therapies could probably be independent of one specific targeted immunotherapy and should instead focus on microbiota composition changes.

Unfortunately, microbiota changes during hormonal therapy remain poorly described. We identified only two studies published in this field. One study collected longitudinal microbiota samples and the other performed cross-sectional microbiota sampling. Hormonal therapies consisted of somatostatin analogs or androgen axis-targeted therapies with ATT or GNRH [31,42]. No explanation is available as to why somatostatin analogs had no influence on human intestinal microbiota. Treatment with ATT resulted in a microbiota with low β-diversity. Both ATT and GNHR therapy were related to significant microbiota composition changes in patients with prostate cancer. However, this cross-sectional study compared patients treated with hormonal therapy with healthy controls but also patients without treatment, resulting in small and heterogeneous groups.

The observations could be explained by the potential influence of androgen axis therapy on bacterial steroid biosynthesis [59]. Assuming that hormonal therapy interacts with the intestinal microbiota involved in steroid/hormone synthesis, this might modulate steroid biosynthesis, thereby affecting systemic hormone levels and therapy efficacy [12,60,61].

### 4.3. Strengths and Limitations

Gut microbiota research is a field with great biological complexity, imposing considerable challenges on the researchers. A strength of particularly the more recent studies is the use of 16S rRNA gene sequencing or even WMGS. These techniques are superior to other microbiota profiling techniques—like qPCR, PCR-DGGE, or FISH—and provide a detailed overview of microbiota composition, with high taxonomic resolution. WMGS even offers the possibility to quantify functional capacity of the gut microbiota.

However, we also identified several limitations, reducing generalization of the results. First of all, it is difficult to compare the different studies under investigation, since sampling time points, study design, and methods used for microbiota profiling were highly heterogeneous. For example, in the study of Gopalakrishnan et al. (2018), baseline sampling took place over a broad range of days. Furthermore, different approaches were used in order to distinguish between responders and non-responders or to quantify treatment response and complications.

Next to the heterogeneity between studies, some studies also suffer from high heterogeneity within the study, due to the inclusion of patients with different cancer types and/or different drugs. This leads to a study population with a high level of heterogeneity and inadequate comparability. Another limitation is the relatively small population size studied in the majority of the studies. Since gut microbiota composition is known to be highly different between individuals [62], greater sample sizes are needed. The problem of small sample sizes gets even worse due to substantial loss to follow up, resulting in small groups to draw conclusions on.

**Figure A1:**
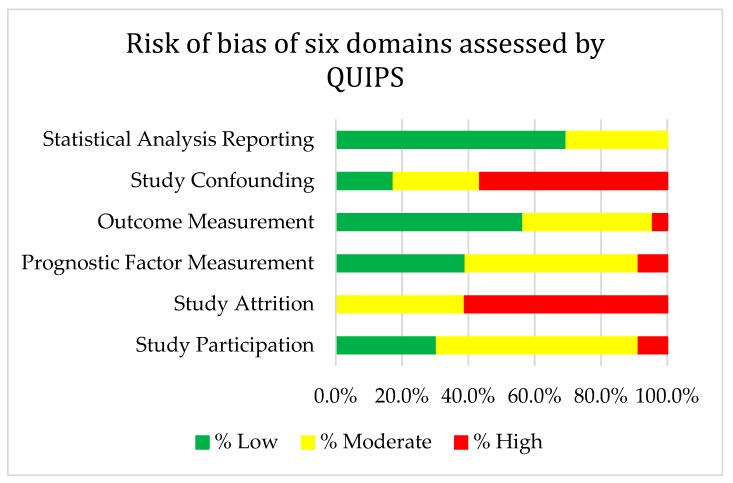
Risk of bias of six domains assessed by QUIPS.

Since gut microbiota composition is influenced by several external factors, the risk of bias is generally high in microbiota research. Strong confounding factors in this field are antibiotic use, age, BMI, and diet [62]. Attention and correction for these confounders was very different between the studies (Appendix A, Table A1 and Figure A1). In most of the studies, the measurement of potential confounders was considered to be insufficient. Particularly, the regularly observed insufficient assessment of and correction for previous antibiotic use might be problematic, since antibiotics have also been shown to be associated with (breast) cancer risk [63]. Besides, it has been recently demonstrated that antibiotics modulate gut microbiota composition and metabolite production as well as key metabolic processes and tumor growth [64]. These findings support the necessity that antibiotic use should be adequately reported in clinical microbiota studies and that patients with previous antibiotic use should be distinguished from patients without use of antibiotics.

Despite the fact that systemic cancer therapy most likely also affects stool consistency and bacterial biomass, changes in these parameters were hardly assessed in the studies under investigation. Vandeputte et al. (2016) showed that stool consistency was strongly associated with microbiota richness as well as with community composition and abundance of specific enterotypes [65]. Consequently, studies neglecting these parameters might imply the risk that reported changes in gut microbiota composition and diversity can be attributed to changes in microbial biomass. Therefore, it is considered to be of great benefit for microbiota research to correct for this strong confounding effect.

## 5. Materials and Methods

### 5.1. Review Questions

Main questions for this review were if:

- Baseline human intestinal microbiota was associated with systemic cancer therapy outcome
- Human intestinal microbiota changed during systemic cancer therapy

### 5.2. Review Search

A thorough systematic literature search was performed using the following databases: Annual review, BioMed Central, Cochrane Library, EBMR, EMBASE, Informa Healthcare, Medline, and PubMed.

By using the Boolean Search Operator, the following query was created: “(((((Microbiota OR microbiome OR “gut microbiota” OR “gut microbiome” OR “intestinal microbiota” OR “intestinal microbiome” OR “gastrointestinal microbiota” OR “gastrointestinal microbiome”)) AND (“cancer treatment” OR “cancer treatments” OR “cancer therapy” OR “cancer therapies” OR “anticancer therapy” OR “anticancer therapies” OR “systemic therapy” OR “systemic therapies” OR chemotherapy OR chemotherapies OR chemotherapeutics OR “hormone treatment” OR “hormone treatments” OR “hormone therapy” OR “hormone therapies” OR immunotherapy OR immunotherapies OR “antineoplastic”)) AND (Cancer OR neoplasm OR neoplasms)) AND (Human OR humans)) NOT (Murine OR mice OR mouse OR rat OR rats)”. No limits were set in any database. Predefined inclusion and exclusion criteria were used for article selection. The last search was performed April 22 2019.

### 5.3. Eligibility Criteria

The systematic search was structured by means of the PICOS acronym (participants, interventions, comparators, outcome measures, study design).

The PICOS criteria were identified as follows:

- Types of participants: human participants with any type of cancer.
- Types of interventions: systemic cancer therapy with chemotherapy, immunotherapy, or hormone therapy.
- Types of comparators: studies comparing baseline and or follow up intestinal microbiota composition in patients starting and/or receiving systemic cancer therapy with either healthy controls, no intervention, follow up samples, and/or therapy outcomes.
- Types of outcome measures: intestinal microbiota associated therapy outcomes and intestinal microbiota composition changes analyzed with any type of detection method.
- Types of study design: observational studies or intervention studies with a control and/or placebo group.

All studies that did not fulfill the PICOS characteristics were excluded. In addition, animal studies, conference papers, abstracts, as well as articles that were not available in full text in Dutch or English were excluded.

### 5.4. Study Selection

**Figure 2:**
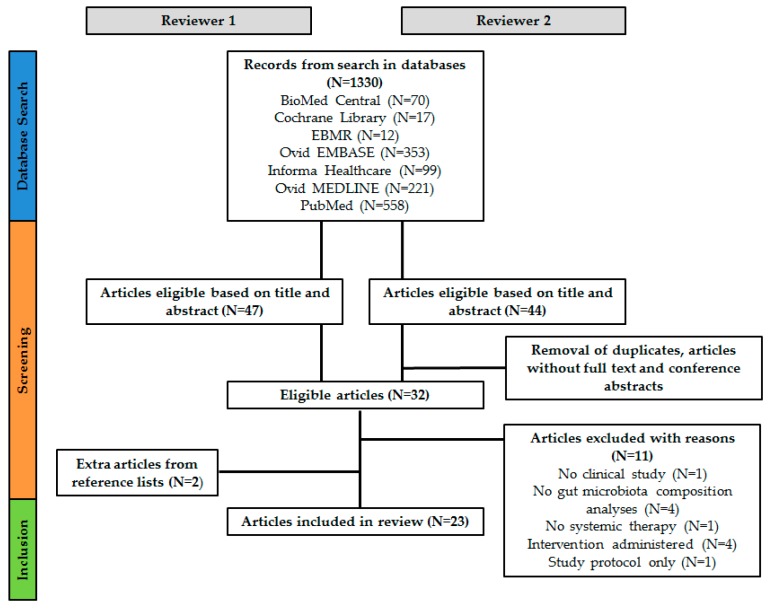
Schematic overview of the article selection procedure.

Two researchers (R.A. and J.Z.) independently examined the databases for eligible articles based on title and abstract. Duplicates, articles without full text available and conference abstracts were removed. With regard to the remaining articles, discrepancies between the two reviewers were discussed until agreement for in- or exclusion was reached. This generated a list of 32 articles. Subsequently, both researchers read the full text of the articles which led to the exclusion of another 11 articles. Additionally, the reference lists of included articles were screened for additional articles and these were included after approval of the second reviewer. Finally, 23 articles were included in the current review. Figure 2 provides an overview of the article selection process.

### 5.5. Data Collection Process

Data extraction was conducted following a data extraction sheet conform Table 1, Table 2 and Table 3. Data extraction was performed in an unblinded and independent manner by the two reviewers (R.A. and J.Z.). Disagreements were discussed and resolved until consensus was reached.

### 5.6. Risk of Bias Assessment

The risk of bias in the individual studies was evaluated with the Quality In Prognosis Studies (QUIPS) tool, which is recommended by the Cochrane Prognosis Methods Group to assess the risk of bias in prognostic studies [66]. The QUIPS tool consisted of the following domains: (1) study participation, (2) study attrition, (3) prognostic factor measurement, (4) outcome measurement, (5) study confounding, (6) statistical analysis and reporting. Based on whether specific criteria were fulfilled or not, the risk of bias per domain was defined as low, moderate, or high (Appendix A, Table A1 and Figure A1). Two reviewers (R.A. and J.Z.) independently assessed the risk of bias and consensus was reached afterwards.

## 6. Future Directions

The rapidly growing number of publications concerning microbiota-based cancer therapy interactions emphasizes the great relevance of the topic. However, evidence obtained in a clinical setting is still limited. Therefore, there is an urgent need for well controlled human studies to further elucidate the role of the gut microbiota in human cancers and to evaluate its potential as therapeutic target. From our perspective, future research should focus on two main aspects:

1. The predictive ability of pre-treatment intestinal microbiota concerning development of complications and response to cancer treatment.
2. The potential use of microbiota-modulating strategies in order to improve cancer therapy outcome.

It would be a breakthrough in cancer therapy if patients at risk of developing complications or having a lower chance of success could be identified in advance, based on their microbiota profile. This would not only facilitate precision medicine but would also give the opportunity to intervene at an early stage by means of microbiota-targeted interventions. Therefore, future research should evaluate the potential of the gut microbiota as a biomarker for therapy success.

Concerning the sampling of feces, we recommend that future research should perform longitudinal sampling, since this provides important information concerning changes over time and is considered superior to cross-sectional comparisons. In addition, it is strongly advised to take the different confounders into consideration for the study design of upcoming studies. In particular, the quality of research will greatly benefit by assessing antibiotic use, BMI, and dietary intake.

Furthermore, there is currently a shortage of studies investigating the functional capacity of the gut microbiome, since most of the studies focus on gut microbiota composition. This lack of knowledge might be filled by future studies measuring microbial metabolites (meta-metabolomics) or gene expression (metatranscriptomics).

In the present review, it also became clear that scientific evidence is particularly scarce in the field of hormonal therapy. Two years ago, our research group started an observational cohort study with longitudinal fecal sample collection to study the microbiota composition before and during hormonal therapy in postmenopausal breast cancer patients receiving aromatase inhibitors or tamoxifen. Specifically, in the aromatase group (*n* = 60), we will study circulating hormonal levels related to the human intestinal microbiota composition and therapy efficacy. In the tamoxifen group (*n* = 60), endoxifen levels and tamoxifen-related human intestinal microbiota changes will be studied. The upcoming results will bridge the knowledge gap and will provide novel insights into hormonal therapy efficacy. In addition, similar longitudinal studies are on-going in CRC and breast cancer patients receiving chemotherapy [67].

Finally, proven interaction of human intestinal microbiota with systemic cancer therapy should lead to the evidence-based design of clinical trials targeting the gut microbiota. Possible strategies would be prebiotics, probiotics, as well as FMT. Currently, several clinical trials using FMT (e.g., NCT03341143) or probiotics (e.g., NCT00197873, NCT03642548, or NCT03705442) are on-going. Results of these studies may reveal the potential of microbiota-targeted interventions in cancer patients, although more fundamental knowledge is likely needed to guide the selection of specific intervention strategies.

## Figures and Tables

**Table 1:** Clinical studies investigating the association between baseline intestinal microbiota composition and systemic cancer therapy outcome and complications.

Study Design					Main Findings	
Study	Cancer Type	*n*	Type of Therapy	Analysis Method	Therapy Outcome	Microbial Outcomes Found to Be Different
**Chemotherapy**						
Galloway-Peña et al. (2017), [21]	AML	*n* = 35	Induction chemotherapy	16S rRNA gene sequencing	Increased risk for infections	↑ intra-patient temporal variability of α-diversity (CV of Shannon index) ↑ *Stenotrophomonas*
Galloway-Peña et al. (2016), [20]	AML	*n* = 34	Induction chemotherapy	16S rRNA gene sequencing	Increased risk for infections	*↓* baseline α-diversity (Shannon index)
Pal et al. (2015), [23]	Metastatic RCC	*n* = 20	VEGF-TKI	16S rRNA gene sequencing	Increased risk to develop diarrhea	↑ *Bacteroides* *↓ Prevotella*
**Immunotherapy**						
Matson et al. (2018), [24]	Metastatic melanoma	*n* = 42	Anti-PD-1 (*n* = 38) Anti-CTLA-4 (*n* = 4)	16S rRNA gene sequencing Metagenomic shotgun sequencing qPCR	Response (*n* = 16)	↑ Bifidobacteriaceae ↑ *Enterococcus faecium* ↑ *Collinsella aerofaciens* ↑ *Bifidobacterium adolescentis* ↑ *Klebsiella pneumoniae* ↑ *Veillonella parvula* ↑ *Parabacteroides merdae* ↑ *Lactobacillus* sp. ↑ *Bifidobacterium longum* *↓ Ruminococcus obeum* *↓ Roseburia intestinalis*
Gopalakrishnan et al. (2018), [25]	Metastatic melanoma	*n* = 43	Anti-PD-1	16S rRNA gene sequencing	Response (*n* = 30)	↑ α-diversity (inverse Simpson score) ↑ between-group β-diversity ↑ Clostridiales ↑ Ruminococcaceae ↑ *Faecalibacterium* *↓* Bacteroidales
		*n* = 25	Anti-PD-1	Metagenomic whole-genome shotgun sequencing	Response (*n* = 14)	↑ *Faecalibacterium* sp. ↑ *Clostridium* sp. ↑ *Clostridiales* *↑ Eubacterium* sp. ↑ *Oscillibacter sp*. ↑ *Ruminococcaceae* *↓ Bacteroides thetaiotaomicron* *↓ Escherichia coli* *↓ Oxalobacter formigenes* *↓ Anaerotruncus colihominis* *↓ Klebsiella variicola*
		*n* = 39	Anti-PD-1	Metagenomic whole-genome shotgun sequencing	Prolonged PFS (*n* = 19)	↑ *Faecalibacterium* *↓* Bacteroidales
Routy et al. (2018), [26]	NSCLC (*n* = 60) RCC (*n* = 40)	*n* = 100	Anti-PD-1	Metagenomic shotgun sequencing	Response	↑ α-diversity (richness) *↑ Akkermansia muciniphila* *↑ Firmicutes* and *4x unclassified* *↑ Eubacterium* sp. *↑ Lachnospiraceae* *↑ Erysipelotrichaceae bacterium* *↑ Cloacibacillus porcorum* *↑ Enterococcus faecium* *↑ Intestinimonas* *↑ 2x unclassified Clostridialis* *↑ Alistipes* *↑ Bacteroides* sp. *↑ Alistipes indistinctus* *↑ Firmicutes bacterium* *↑ Prevotella* *↓ Prevotella* *↓ Clostridium* sp. *↓ unclassified Firmicutes* *↓ Prevotella* sp. *↓ Clostridiales* *↓ Clostridium bolteae* *↓ Firmicutes bacterium* *↓Closteridiales bacterium* *↓ Blautia* *↓ Bacteroides clanus* *↓ Proteobacteria* *↓ Bacteroides nordii* *↓ Parabacteroides distasonis*
	NSCLC + RCC	*n* = 78	Anti-PD-1	Metagenomic shotgun sequencing	PFS > 3 months	*↑ unclassified Firmicutes 6x* *↑ Eubacterium* sp. *↑ Alistipes 2x* *↑ Akkermansia muciniphila* *↑ Intestinimonas* *↑ Bacteroides nordii* *↑ Bacteroides xylanisolvens* *↑ Blautia* *↑ Lachnospiraceae* *↑ Firmicutes bacterium* *↑ Firmicutes* *↑ unclassified Clostridiales 2x* *↑ Clostridialis* *↑ Ruminococcaceae* *↑ Clostridium* sp. *↑ Flavonifractor* *↑ Bacteroides caccae* *↑ unclassified Ruminococcaceae* *↑ Ruminococcus* sp. *↓ unclassified Clostridialis* *↓ Parabacteroides distasonis* *↓ Firmicutes bacterium* *↓ Clostridiales* *↓ Clostridialis VE202-14* *↓ Anaerotruncus colihominis* *↓ Lachnospiraceae* *↓ Erysipelotrichaceae*
Chaput et al. (2017), [27]	Metastatic melanoma	*n* = 26	Ipilimumab	16S rRNA gene sequencing	Colitis and good response	↑ Firmicutes *↓* Bacteroidetes
					↑ PFS ↑ OS ↑ % clinical benefit	↑ *Faecalibacterium* ↑ Firmicutes ↑ unclassified *Ruminococcaceae* ↑ *Clostridium XIVa* ↑ *Blautia*
Frankel et al. (2017), [28]	Metastatic/ unresectable melanoma	*n* = 39	Ipilimumab, Nivolumab Ipilimumab + Nivolumab Pembrolizumab	Metagenomic shotgun sequencing	Response (*n* = 24)	↑ *Bacteroides caccae* ↑ *Streptococcus parasanguinis*
		*n* = 24	Ipilimumab + Nivolumab	Metagenomic shotgun sequencing	Response (*n* = 16)	↑ *Faecalibacterium prausnitzii* ↑ *Holdemania filiformis* ↑ *Bacteroides thetaiotamicron*
		*n* = 13	Pembrolizumab	Metagenomic shotgun sequencing	Response (*n* = 6)	↑ *Dorea formicigenerans*
Dubin et al. (2016), [29]	Metastatic Melanoma	*n* = 34	Ipilimumab	16S rRNA gene sequencing	Colitis free	↑ Bacteroidaceae ↑ Bacteroides ↑ Barnesiellaceae ↑ Barnesiellaceae unclassified ↑ Rikenellaceae ↑ Rikenellaceae unclassified ↑ Bacteroidetes ↑ Bacteroidia ↑ Bacteroidales ↑ *Bacteroidetes*
**Other**						
Montassier et al. (2016), [22]	Non-Hodgkin lymphoma	*n* = 28	HSCT	16S rRNA high-throughput DNA sequencing	Increased risk to develop bloodstream infections	↑ Erysipelotrichaceae ↑ *Veillonella* *↓* α-diversity (phylogenetic diversity, observed species, Chao1 & Shannon indices) *↓* Barnesiellaceae ↑ *Butyricimonas* *↓* Christensenellaceae *↓ Faecalibacterium* *↓ Oscillospira* *↓ Christensenella* *↓ Dehalobacterium* *↓ Desulfovibrio* *↓ Sutterella* *↓ Oxalobacter* *↓* Coriobacteriaceae

↑: Increase, *↓*: Decrease, AML: Acute myeloid leukemia; CV: coefficient of variation; RCC: renal cell carcinoma; PFS: progression-free survival; OS: overall survival; NSCLC: non-small cell lung cancer; HSCT: hematopoietic stem cell transplantation.

**Table 2:** Clinical studies assessing intestinal microbiota changes during systemic cancer therapy by longitudinal sampling.

Study Design						Main Findings
Study	Type of Cancer	*n*	Type of Therapy	Sampling Time Points	Method Used for Microbiota Analysis	Effects of Therapy on Microbiota
**Chemotherapy**						
Galloway-Peña et al. (2017), [21]	AML	*n* = 35	Induction chemotherapy	Baseline: before or within first 24h of chemotherapy; Follow-up: every 96h until neutrophil recovery	16S rRNA gene sequencing	↑ intra-patient temporal variability of α-diversity (CV of Shannon) ↑ *Staphylococcus* ↑ *Streptococcus* ↑ *Akkermansia* ↑ *Subdilogranulum* ↑ *Pseudobutyrivibrio*
Sze et al. (2017), [34]	CRC	*n* = 26	12 surgery 9 surgery + chemotherapy 5 surgery + chemotherapy + radiation	Before and after treatment	16S rRNA gene sequencing	Change in community structure Shift towards healthy microbiota
Galloway-Peña et al. (2016), [20]	AML	*n* = 34	Induction chemotherapy	Baseline: before therapy; Follow-up: every 96 h until neutrophil recovery	16S rRNA gene sequencing	↑ *Lactobacillus* ↓ α-diversity (Shannon index) ↓ *Blautia*
Rajagopala et al. (2016), [15]	ALL	*n* = 28	Chemotherapy	(1) Before therapy, (2) during induction chemotherapy (3) during consolidation chemotherapy (4) during maintenance chemotherapy	16S rRNA gene sequencing	↑ α-diversity (Shannon index)
Montassier et al. (2015), [33]	Non-Hodgkin’s lymphoma	*n* = 28	Chemotherapy	Baseline: before chemotherapy; Follow-up: 7 days later	16S rRNA gene sequencing	↑ Proteobacteria ↑ *Citrobacter* ↑ *Klebsiella* ↑ *Enterococcus* ↑ *Megasphaera* ↑ *Parabacreroides* ↓ α-diversity (Faith’s phylogenetic diversity, observed species) ↓ Firmicutes ↓ Actinobacteria ↓ *Ruminococcus* ↓ *Oscillospira* ↓ *Blautia* ↓ *Lachnospira* ↓ *Roseburia* ↓ *Dorea* ↓ *Coprococcus* ↓ *Anaerostipes* ↓ *Clostridium* ↓ *Collinsella* ↓ *Adlercreutzia* ↓ *Bifidobacterium*
Montassier et al. (2014), [6]	Non-Hodgkin’s lymphoma	*n* = 8	Chemotherapy	Baseline: before chemotherapy; Follow-up: 1 week after chemotherapy	16S rRNA gene pyrosequencing/dHPLC	↑ Bacteroidetes ↑ Proteobacteria ↑ *Bacteroides* ↑ *Escherichia* ↓ α-diversity (OTUs, Chao index, Shannon index) ↓ Firmicutes ↓ Actinobacteria ↓ *Blautia* ↓ *Faecalibacterium* ↓ *Roseburia* ↓ *Bifidobacterium*
Stringer et al. (2013), [37]	Breast cancer, gastrointestinal cancer	*n* = 10	Chemotherapy (FOLFOX4, FOLFOX6, FOLFIRI, capecitabine)	(1) Before chemotherapy (2) Day 2 of chemotherapy (3) Day 5 (4) Day 10	Bacterial growth tests with selective media, real-time PCR	↑ *E.coli* ↑ *Lactobacillus spp*. (until day 5, then decrease)
Dörffel et al. (2012), [31]	NET	*n* = 13	Chemotherapy	Before and during therapy	FISH	↑ *Faecalibacterium prausnitzii* (midgut NET only)
Zwielehner et al. (2011), [32]	Different types of cancer	*n* = 17	Chemotherapy	(1) Before chemotherapy (2) Day 1–4 after chemotherapy (5) Day 5–9 after chemotherapy	qPCR/PCR-DGGE	↓ *Bacteroides* ↓ *Bifidobacteria* ↓ *Clostridium* cluster *IV* ↓ *Clostridium* cluster *XIVa*
		*n* = 2	Chemotherapy	(1) Before chemotherapy (2) Day 1–4 after chemotherapy	High throughput sequencing	↑ *Enterococcus faecium* ↑ *Clostridium difficile* ↑ Peptostreptococcaceae ↓ *Faecalibacterium prausnitzii* ↓ Lactobacilli ↓ *Veillonella* spp. ↓ Bifidobacteria ↓ *E.coli/Shigella*
Wada et al. (2010), [38]	Different types of cancer	*n* = 23	Chemotherapy	(1) Before chemotherapy (2) Within 24 h after initiation (3) Once weekly	Bacterial cultures (*n* = 3)	↑ Enterobacteriaceae
Van Vliet et al. (2009), [39]	Pediatric AML	*n* = 9	Chemotherapy	(1) Day 2 of chemotherapy (2) Day 11 of chemotherapy (3) ≥6 weeks after treatment	PCR-DGGE	↓ α-diversity
**Immunotherapy**						
Routy et al. (2018), [26]	NSCLC (*n* = 15) RCC (*n* = 17)	*n* = 32	Anti-PD-1	(1) Before treatment (2) After 2nd injection (1 month) (3) After 4th injection (2 months) (4) After 12th injection (6 months	Metagenomic shotgun sequencing	↑ α-diversity (Richness) ↑ *Candidatus Alistipes marseilloanorexicus* ↑ *Clostridium scindens* ↑ *Eubacterium* sp. ↑ *Clostridium* sp. ↑ *Streptococcus salivarius* ↑ *Clostridiales* ↑ *Eubacterium eligens*
Chaput et al. (2017), [27]	Metastatic melanoma with colitis	*n* = 7	Ipilimumab	At baseline and at the time of colitis occurrence	16S rRNA gene sequencing	↓ α-diversity (Shannon index) ↓ *Ruminococcus* ↓ *Lachnospiracea incertae sedis* ↓ *Blautia* ↓ *Clostridium IV* ↓ *Eubacterium* ↓ *unclassified Lachnospiraceae* ↓ *Pseudoflavonifracto* ↓ Butyrate producing bacterium L2-21 ↓ *Ruminococcus bromii* ↓ *Blautia obeum 1-33* ↓ *Eubacterium coprostanoligenes HL* ↓ *Clostridium clostridioforme LCR24* ↓ *Alistipes spe 627* ↓ *Blautia obeum* ↓ Butyrate producing bacterium PH08AY04 ↓ *Clostridium leptum DSM 753T* ↓ *Bacterium ASF500* ↓ *Clostridium sp JC3* ↓ *Rumen bacterium 2-293-25* ↓ *Bacterium ic* ↓ Butyrate producing bacterium M21-2 ↓ Unidentified bacterium CCCM23 ↓ Unidentified bacterium CCCM41 ↓ *Ruminococcus bromii L2-63* ↓ *Clostridiales bacterium JN18-V41*
Vetizou et al. (2015), [41]	Metastatic melanoma	*n* = 18	Ipilimumab	See Chaput et al. (2017)	16S rRNA gene sequencing	↑ *Bacteroides salyersiae* ↑ *Bacteroides acidifaciens* ↑ *Bacteroides uniformis* ↓ *Prevotella copri* ↓ *Bacteroides* sp. ↓ *Barnesiella intestinohominis* ↓ *Parabacteroides distasonis*
Dörffel et al. (2012), [31]	Midgut NET	*n* = 11	Interferon alpha-2b	Before and during therapy	FISH	↑ *Faecalibacterium prausnitzii*

↑: Increase, ↓:Decrease, AML: acute myeloid leukemia; CRC: colorectal cancer; ALL: acute lymphoblastic leukemia; dHPLC: denaturing high-performance liquid chromatography; NET: neuroendocrine tumor; PCR-DGGE: polymerase chain reaction denaturing gradient gel electrophoresis; RCC: renal cell carcinoma; NSCLC: non-small cell lung cancer,; FISH: fluorescent in situ hybridization.

**Table 3:** Clinical studies assessing intestinal microbiota changes during systemic cancer therapy by cross-sectional sampling.

Study Design						Main Findings
Study	Type of Cancer	Type of Therapy	*n* Cases	*n* Controls	Method Used for Microbiota Analysis	Effects of Therapy on Microbiota
**Chemotherapy**						
Youssef et al. (2018), [35]	Gastrointestinal cancer	Chemotherapy and/or radiotherapy	*n* = 20 (treated patients)	Non-treated patients: *n* = 43	16S rRNA gene sequencing	↑ Lactobacillaceae ↑ *Lactobacillus*
				Healthy controls: *n* = 13	16S rRNA gene sequencing	↓ Bifidobacteriaceae ↓ *Ruminiclostridium* ↓ *Lachnoclosteridium* ↓ *Oscillibacter*
Deng et al. (2018), [36]	CRC	Oxaliplatin + tegafur	*n* = 14	*n* = 33	16S rRNA gene sequencing	↑ *Veillonella* ↑ *Veillonella dispar* ↑ *Prevotella copri* ↑ *Bacteroides plebeius*
Stringer et al. (2013), [37]	CRC, breast cancer, laryngeal cancer, esophageal cancer, melanoma	Chemotherapy	*n* = 16	*n* = 2	Bacterial growth tests with selective media, real-time PCR	↑ *Escherichia coli* ↑ *Staphylococcus* spp. ↓ *Lactobacillus* spp. ↓ *Bacteroides spp*. ↓ *Bifidobacterium* spp. ↓ *Enterococcus* spp.
Van Vliet et al. (2009), [39]	Pediatric AML	Chemotherapy	*n* = 9	*n* = 11	FISH	↑ Enterococci ↓ total number of bacteria ↓ *Bacteroides* ↓ *Clostridium* cluster *XIVa* ↓ *Faecalibacterium prausnitzii* ↓ *Bifidobacterium* ↓ Streptococci
**Hormonal Therapy**						
Sfanos et al. (2018), [42]	Prostate cancer	ATT/GNRH	ATT: *n* = 9 GNRH: *n* = 5	*n* = 16 (no medication)	16S rDNA sequencing	Smallest β-diversity within ATT compared to GNRH and controls Greatest β-diversity between ATT and no medication
		ATT	*n* = 9	*n* = 16 (no medication)	16S rDNA sequencing	↑ *Akkermansia muciniphila* ↑ Ruminococcaceae ↑ *Blautia wexlerae* ↑ *Clostridium oroticum* ↑ *Lachnospiraceae_Clostridium_XlVa* ↑ *Robinsoniella peoriensis* ↑ *Anaerococcus tetradius* ↑ *Bacteroides stercoris* ↑ Verrucomicrobiaceae ↑ Lachnospiraceae ↑ *Clostridiales insertae sedis XIII* ↑ Staphylococcaceae ↑ Bacillales ↑ Aerococcaceae ↑ Selenomonadales ↓ Clostridiales ↓ Brevibacteriaceae ↓ Erysipelotrichaceae ↓ Streptococcaceae ↓ Clostridiales_unassigned ↓ Prevotellaceae
		ATT	*n* = 9	*n* = 16 (no medication) *n* = 5 (GNRH)	qPCR	↑ *Akkermansia muciniphila*
		GNRH	*n* = 5	*n* = 16 (no medication)	16S rDNA sequencing	↑ *Blautia wexlerae* ↑ *Clostridium oroticum* ↑ *Anaerococcus tetradius* ↑ Lachnospiraceae ↑ Staphylococcaceae ↑ Aerococcaceae ↑ Selenomonadales

↑: Increase; ↓: Decrease; AML: acute myeloid leukemia; CRC: colorectal cancer; FISH: fluorescent in situ hybridization; ATT: androgen receptor axis-targeted therapy; GNRH: gonadotropin-releasing hormone.

## Abbreviations

5-FU	5-fluorouracil
AML	Acute myeloid leukemia
ALL	Acute lymphoblastic leukemia
ATT	Androgen receptor axis-targeted therapies
BSI	Bloodstream infections
CPIs	Checkpoint inhibitors
CRC	Colorectal cancer
CTLA-4	Cytotoxic T-lymphocyte-associated protein 4
CV	Coefficient of variation
dHPLC	Denaturing high-performance liquid chromatography
FISH	Fluorescent in situ hybridization
FMT	Fecal microbiota transplantation
GNRH	Gonadotropin-releasing hormone
GPCR	G protein-coupled receptor
I	Ipilimumab
IN	Ipilimumab + nivolumab
LDA	Linear discriminant analysis
N	Nivolumab
NET	Neuroendocrine tumors
NSCLC	Non-small cell lung cancer
OTU	Operational taxonomic unit
OS	Overall survival
P	Pembrolizumab
PCA	Principal component analysis
PCoA	Principal coordinate analysis
PCR-DGGE	Polymerase chain reaction denaturing gradient gel electrophoresis
PD-1	Programmed cell death protein 1
PFS	Progression free survival
PICOS	Participants, interventions, comparators, outcome measures, study design
qPCR	Quantitative polymerase chain reaction
QUIPS	Quality In Prognosis Studies
RECIST	Response evaluation criteria in solid tumors
RCC	Renal cell carcinoma
SCFA	Short-chain fatty acids
WMGS	Whole metagenome sequencing

## Appendix A

**Table A1:** Risk of bias of included studies assessed by Quality in Prognosis Studies tool (QUIPS).

Article	Study Participation	Study Attrition	Prognostic Factor Measurement	Outcome Measurement	Study Confounding	Statistical Analysis and Reporting
Deng (2018), [36]		NA				
Gopalakrishnan (2018), [25]		NA				
Matson (2018), [24]		NA				
Routy (2018), [26]						
Sfanos (2018), [42]		NA				
Youssef (2018), [35]		NA				
Chaput (2017), [27]						
Frankel (2017), [28]						
Galloway Pena (2017), [21]						
Sze (2017), [34]						
Dubin (2016), [29]						
Rajagopala (2016), [15]						
Galloway-Pena (2016), [20]						
Montassier (2016), [22]						
Vetizou (2015), [41]						
Montassier (2015), [33]						
Pal (2015), [23]						
Montassier (2014), [6]						
Stringer (2013), [37]						
Zwielehner (2011), [32]						
Dörffel (2011), [31]						
Wada (2010), [38]						
Van Vliet (2009), [39]						

## Appendix B

**Table A2:** Definition of terms used in microbiota research.

α-diversity	Number and evenness of distribution of taxa within a given sample
β-diversity	The difference in diversity of taxa from one sample to another, i.e., the number of taxa that are not the same (or not similarly distributed) in two different samples.
16S rRNA gene	Marker gene for bacterial identification, containing evolutionary conserved universal as well as variable regions
Operational taxonomic unit (OTU)	Cluster of nearly-identical sequences (e.g., 97% similarity), often used in microbiota research instead of ‘species’
16S rRNA gene sequencing	Sequencing of the 16S rRNA marker gene
Metagenomic sequencing	Sequencing of the entire metagenome (all the genetic material in a sample), also allowing analysis of the functional capacity of the microbiome
